# Editorial

**DOI:** 10.34763/jmotherandchild.20212502.edit.2021_25_02

**Published:** 2022-04-01

**Authors:** Marcin Czech

**Affiliations:** 1 Department of Pharmacoeconomics, Institute of Mother and Child, Warsaw, Poland

## COVID-19: The End or the Beginning?

The issue 2021;25(2) of the *Journal of Mother and Child* is mainly focused on COVID-19 in pregnant women and paediatric populations. It also covers an interesting problem of viral co-infections: coronaviral and caused by HIV, as well as a mixed one resulted from the simultaneous appearance of tuberculosis and SARS- CoV-2. With the fourth wave of infections, increasing number of cases, excess mortality directly or indirectly linked with the disease, limited healthcare sector resources, especially in pulmonology departments and intensive care units, and still unknown long-term consequences, this subject deserves a lot of attention.

It is well known to most people, both healthcare professionals and lay people, that corona virus disease (COVID-19) is an infectious disease caused by the novel virus that may lead to a severe acute respiratory syndrome (SARS Cov-2). Paediatric patients infected mainly at schools and kindergartens, but also in households, account for less than 5% of total COVID-19 patients worldwide. Their symptoms are usually mild or nonexistent. For these reasons, studies involving children are rare. As a result, there are inadequate data about COVID-19 infection in this subpopulation.

One of the articles from this issue is a systematic review looking at epidemiology, transmission, clinical characteristics, diagnosis, and management of COVID-19 infection in paediatric populations. According to one of the selected studies, 13% of children who were diagnosed with COVID-19 were asymptomatic. Epidemiological extrapolation to the general population is challenging, since the likelihood of asymptomatic individuals being tested is lower, which is one of the reasons potentially promoting the spread of the disease. Children could also be suffering from other infections, such as the ones caused by RSV, recently very common in Poland, which may render the detection of COVID-19 difficult. It seems that not only in adults but also in children, COVID-19 infection prognosis could be worsened by weight gain and cigarette smoke exposure. A diagnostic challenge is that 80% of children who were COVID-19 positive per tests done through nasopharyngeal swabs continued to show repeated positive results for real-time reverse transcriptase-polymerase chain reaction (RT-PCR) on rectal swabs, even after their nasopharyngeal swabs turned out to be negative. Among the paediatric patients, 12.9% were asymptomatic, 43.1% had mild illness, 41% were moderately ill, 2.5% had a severe illness, and 0.4% were critical. Paediatric subjects having a confirmed COVID-19 diagnosis exhibited slower progression compared with a general population, which indicates that the illness caused by COVID-19 could be less severe compared with other respiratory disorders. The minimum time between infection onset and the confirmed diagnosis was 48 hours. What is interesting, for paediatric patients having a severe or critical form of the illness, the incidence was 10.6% for infants less than 12 months of age, while it was 7.3%, 4.2%, and 4.1% for children of 1–5 years, 6–10 years, and 11–15 years, 3.0% over 15 years respectively. These numbers suggest that infants are more susceptible to COVID-19 infection when compared with older children.

So far, there has been no proof of an effective and safe drug against COVID-19, neither for adults nor for children. Various antivirals and immunomodulatory medications, such as interferon lopinavir-ritonavir, chloroquine, azithromycin, remdesivir, tocilizumab, and convalescent plasma therapy were tried with no conclusive outcome.

The majority of the publications described in the systematic review have not been peer-reviewed and the most of the studies were from China. For a better understanding of COVID-19 infection in children, research with a wider regional focus should be planned and implemented – we are at the beginning of a knowledge collection pathway. What is more, data regarding transmission are still inadequate and the management of COVID-19 infection in children is not specific.

Data regarding pregnant women and newborns are even more scarce. As physiological changes occurring during pregnancy can have a positive or negative effect on the disease progression, the objective of one of the studies was to evaluate the maternal and neonatal outcomes in pregnant women with COVID-19 compared to pregnant women without COVID-19. According to the authors, there was no significant effect of the infection on maternal and foetal outcomes. Further studies and longterm follow-up are needed to look for any delayed effects on the babies and mothers. Out of 92 COVID-positive pregnant women, only 3 babies tested positive for COVID-19, so vertical transmission probability seems to be low. These results confirm the findings already known from the literature. Overall, all babies were healthy and the majority of mothers were discharged without any complications.

Regarding co-infections, the COVID-19 pandemic has stopped or delayed many essential health care services, including HIV prevention, testing, and treatment. It has imposed serious challenges for children, adolescents, and pregnant women living with HIV worldwide. Addressing pregnant women and children living with HIV should be made a priority by providing them care, including antiretroviral therapy and testing of neonates delivered to infected mothers, as they constitute the most vulnerable group during the paired pandemic. They should be taken care of by optimising funding and strengthening the health care system at all levels.

One can find guidelines addressing management of COVID-19 and pulmonary tuberculosis. There are no guidelines, however, on how to manage COVID-19 and tuberculosis co-infection in pregnancy. During pregnancy treated as a physiological state, the use of various drugs and their outcomes are altered. In this issue readers can find a case study of COVID-19 and tuberculosis co-infection in pregnancy which were managed successfully.

Despite the fact of constantly growing evidence on diagnosis, treatment and management of COVID-19 patients, including the massive vaccination programme and making vaccines available for younger citizens, there is still room for new studies focusing on different aspects of this still-new medical condition.

